# Selective adrenergic alpha2C receptor antagonist ameliorates acute phencyclidine-induced schizophrenia-like social interaction deficits in rats

**DOI:** 10.1007/s00213-018-5130-2

**Published:** 2018-12-10

**Authors:** Katja Savolainen, Jouni Ihalainen, Aaro J. Jalkanen, Markus M. Forsberg

**Affiliations:** School of Pharmacy, University of Eastern Finland, P.O. Box 1627, FI-70211 Kuopio, Finland

**Keywords:** α_2C_ adrenergic receptor antagonist, α_7_ nicotinic acetylcholine receptor partial agonist, Atypical antipsychotics, Rat, Schizophrenia, Social interaction deficit

## Abstract

**Rationale:**

Social withdrawal is a core feature of the negative symptoms of schizophrenia. Currently available pharmacotherapies have only limited efficacy towards the negative symptoms, i.e., there is a significant unmet medical need in the treatment of these symptoms.

**Objective:**

We wanted to confirm whether selective adrenergic α_2C_ receptor (AR) antagonist therapy could ameliorate acute phencyclidine (PCP)-induced schizophrenia-like social interaction deficits in rats, and to compare the effects of an α_2C_ AR antagonist to another putative therapeutic alternative, an α_7_ nicotinic acetylcholine receptor (nAChR) partial agonist, as well against three commonly used atypical antipsychotics.

**Methods:**

Here, we used acute PCP administration and modified a protocol for testing social interaction deficits in male Wistar rats and then used this model to compare the effects of an α_2C_ AR antagonist (ORM-13070 0.3 and 1.0 mg/kg s.c.) with an α_7_ nAChR partial agonist (EVP-6124 0.3 mg/kg s.c.) and three atypical antipsychotics (clozapine 2.5 mg/kg i.p., risperidone 0.04 and 0.08 mg/kg s.c., olanzapine 0.125 and 0.5 mg/kg s.c.) on social interaction behavior.

**Results:**

Acute PCP (1.5 mg/kg s.c.) produced robust and reproducible deficits in social interaction behavior without affecting locomotor activity. The selective α_2C_ AR antagonist significantly ameliorated PCP-induced social interaction deficits. In contrast, neither the partial α_7_ nAChR agonist nor any of the three atypical antipsychotics were able to reverse the behavioral deficits at the selected doses.

**Conclusion:**

Our findings confirm that α_2C_ AR antagonism is a potential mechanism for the treatment of the negative symptoms of schizophrenia.

## Introduction

Schizophrenia is a severe psychiatric disorder affecting approximately 1% of the population. Its symptoms consist of several domains, including positive symptoms (e.g., hallucinations, delusions, incoherence in speech and behavior), negative symptoms (e.g., deficits in social interaction, blunted affect, amotivation, anhedonia), and cognitive deficits (e.g., impairments in attention, memory, and executive functions). Social withdrawal is a core feature of the negative symptoms and it emerges during the early prodromal stage, persisting throughout the course of the illness (Gururajan et al. 2010; Wilson and Koenig 2014). Currently available pharmaco- and psycho-therapies mainly affect the positive symptoms of schizophrenia. Clearly, there is a significant unmet medical need in the treatment of its negative symptoms as both classical and atypical antipsychotics have shown only limited efficacy in their alleviation (Kirkpatrick et al. 2006; Hanson et al. 2010; Sarkar et al. 2015).

Several lines of research are ongoing to establish novel treatment concepts and drug targets for the negative symptoms of schizophrenia. Currently, many potential drug targets are under evaluation, with one of the most interesting being modulation of the adrenergic α_2C_ receptor (AR) function. The α_2C_ ARs have been associated with the treatment of schizophrenia in studies with transgenic mouse models (Scheinin et al. 2001; Svensson 2003), and the recent discovery of α_2_ AR subtype selective compounds has allowed more in-depth investigations on the effects of the α_2C_ AR in different preclinical models, and recently also in humans (Uys et al. 2017). In preclinical schizophrenia models, the α_2C_ AR antagonists have shown some promising antipsychotic-like efficacy against acute phencyclidine (PCP)-induced prepulse inhibition (Sallinen et al. 2007, 2013a) and social interaction deficits in rats (Sallinen et al. 2013a). In addition to α_2C_ AR, the α_7_ nicotinic acetylcholine receptor (nAChR) has been considered as one of the most promising schizophrenia drug targets, this being partly attributable to the evidence of dysfunctional nAChRs in schizophrenia (for a review, see Wallace and Bertrand 2015). The α_7_ nAChR agonists and positive allosteric modulators (PAMs) have been reported to alleviate the negative and cognitive symptoms associated with schizophrenia in acute ketamine-induced rat models (Nikiforuk et al. 2016; Potasiewicz et al. 2017) and subchronic PCP mouse models (Pedersen et al. 2014). Furthermore, α_7_ nAChR partial agonists have shown efficacy against the negative symptoms also in schizophrenic patients as an add-on therapy with antipsychotics, although the main focus of those studies was on cognitive functions (Freedman et al. 2008; Lieberman et al. 2013; Keefe et al. 2015).

Preclinical development of novel pharmacotherapies for schizophrenia strongly relies on the use of valid animal models. The social interaction test, where two unfamiliar rodents are placed in a test environment and the time spent in social interaction behaviors is measured (File and Hyde 1978), is commonly utilized as a preclinical model for social functioning (Wilson and Koenig 2014), a characteristic often disturbed in schizophrenia. Psychotomimetic agents, such as *N*-methyl-D-aspartate (NMDA) receptor antagonists, PCP, and ketamine, are able to induce all domains of schizophrenia-like symptoms in healthy individuals as well as exacerbating the symptoms of patients with schizophrenia (Luby et al. 1959; Cohen et al. 1962; Itil et al. 1967; Krystal et al. 1994). NMDA antagonists interfere with social interaction behavior also in rodents (Gururajan et al. 2010; Wilson and Koenig 2014). Thus, NMDA antagonists are considered to possess good face validity as pharmacological models of schizophrenia, and PCP is one of the most widely used compounds for induction of schizophrenia-like symptoms in rodents. Nonetheless, both subchronic (followed by a drug-free period) (e.g., Sams-Dodd 1996; Pedersen et al. 2014) and acute (e.g., Corbett et al. 1995; Sams-Dodd 1997; Bruins Slot et al. 2005; Sallinen et al. 2013a) PCP administrations have been used to induce social interaction deficits. Acute PCP is typically administered either as a single dose (e.g., Corbett et al. 1995; Boulay et al. 2004; Sallinen et al. 2013a) or combined with a 2- to 4-day pretreatment period to adapt animals to the nonspecific effects of PCP (e.g., Sams-Dodd 1997; Pouzet et al. 2002; Bruins Slot et al. 2005). However, it has also been postulated that this kind of pretreatment is unnecessary when low PCP doses are being used (Boulay et al. 2004). Therefore, an acute low dose PCP administration protocol could provide a straightforward method to screen for the efficacy of novel drug candidates.

The goal of the present study was to confirm whether selective α_2C_ AR antagonist therapy could ameliorate acute PCP-induced schizophrenia-like social interaction deficits in rats. Furthermore, we compared the effects of an α_2C_ AR antagonist to another putative therapeutic alternative, an α_7_ nAChR partial agonist, as well against three commonly used atypical antipsychotics clozapine, risperidone, and olanzapine, which all possess α_2C_ AR antagonistic properties. To achieve these goals, we first modified a simple and straightforward social interaction test protocol in rats based on an acute PCP administration to evoke schizophrenia-like social interaction deficits.

## Materials and methods

### Experimental animals

Male Wistar rats (RccHan:WIST, Laboratory Animal Centre, University of Eastern Finland, Kuopio, Finland, and Harlan Laboratories, The Netherlands and USA; age 10–11 weeks), were used in the social interaction test. The rats were housed in groups of two in stainless steel cages (285 × 485 × 200 mm) under controlled laboratory conditions with 12:12 h light/dark cycle (lights on at 7.00 a.m., temperature 21 ± 2 °C, relative humidity 55 ± 15%). Food (2016S Teklad, Harlan Laboratories, Indianapolis, IN, USA) and water were available ad libitum except during the social interaction test session. The animal testing was performed during the light phase of the day (between 8.00 a.m. and 5.00 p.m.). All experiments were performed in accordance with European Union guidelines (Directive 2010/63/EU and guidelines 2007/526/EC) and approved by the National Animal Experiment Board of Finland.

### Drugs and treatments

Phencyclidine hydrochloride (PCP; 1-(1-Phenylcyclohexyl) piperidine hydrochloride) and clozapine were purchased from Tocris Bioscience (Bristol, UK). Olanzapine and risperidone were purchased from Sigma-Aldrich (St. Louis, MO, USA). An α_7_ nAChR partial agonist EVP-6124 hydrochloride (also known as encenicline hydrochloride; (*R*)-7-chloro-*N*-(quinuclidin-3-yl)benzo[b]thiophene-2-carboxamide hydrochloride) was purchased from MedChem Express (Princeton, NJ, USA). An α_2C_ AR antagonist ORM-13070 (1-[(S)-1-(2,3-dihydrobenzo[1,4]dioxin-2-yl)methyl]-4-(3-methoxymethylpyridin-2-yl)-piperazine; C_20_H_25_N_3_O_3_; MW 355.44) was provided by Orion Pharma (Espoo, Finland). ORM-13070 is a brain penetrating and highly selective α_2C_ antagonist (binding affinity for α_2C_ over α_2A_ is over 28), which has been screened for binding to more than 100 other potential receptors and targets; on them, ORM-13070 displayed either weak or no activity (Arponen et al. 2014). A radiolabeled form (^11^C-ORM-13070) has been used as a PET tracer also in human subjects (Luoto et al. 2014; Lehto et al. 2015a, b, c, 2016).

PCP and EVP-6124 were dissolved in physiological saline (doses refer to the hydrochloride form). Clozapine, risperidone, and olanzapine were dissolved in physiological saline with a minimum amount of 0.1 M HCl. ORM-13070 was dissolved in a mixture of 15% polyethylene glycol 400 and 85% Glucosteril 50 mg/ml. The pH of the solution was adjusted to 4–5 with 1 M HCl. PCP (1.15 and 1.5 mg/kg s.c.), risperidone (0.04 and 0.08 mg/kg s.c.) and olanzapine (0.125 and 0.5 mg/kg s.c.) with their corresponding vehicle solutions were administered in a volume of 5 ml/kg; clozapine (2.5 mg/kg i.p.) and EVP-6124 (0.3 mg/kg s.c.) in a volume of 2 ml/kg; and ORM-13070 (0.3 and 1.0 mg/kg s.c.) in a volume of 1 ml/kg. The PCP dose range for the dose optimization experiment was selected on the basis of the literature (Corbett et al. 1995; Sams-Dodd 1996; Boulay et al. 2004; Sallinen et al. 2013a), taking into account the fact that a dose of 2 mg/kg already affects locomotor activity and induces stereotypical behavior and ataxia in rats (Castellani and Adams 1981; Sams-Dodd 1996; Boulay et al. 2004). The doses for clozapine (Corbett et al. 1995; Maehara et al. 2011), risperidone (Sams-Dodd 1997), and olanzapine (Corbett et al. 1995; Sallinen et al. 2013a) were chosen on the basis of previous rat studies with NMDA antagonists. The dose selection for EVP-6124 was based on the published literature (Prickaerts et al. 2012; Pedersen et al. 2014; Huang et al. 2014) and for ORM-13070 on our pilot experiments.

### Social interaction test

The experimental animals had at least 1 week to acclimatize to the animal facilities prior to the testing procedure. The rats were adapted to handling on three times (separation, weighing, marking), and transferred into single cages 4–7 days before testing to increase the social interaction behavior (Niesink and van Ree 1982). The rats were randomly assigned into treatment groups. An open field arena (600 × 600 × 400 mm, illumination at the floor level 55–65 lx; Samplastic Oy, Kuopio, Finland), made from gray polyvinyl chloride, was used in the social interaction test. A digital video camera was mounted above each of the four arenas used in the study. On the test day, a pair of unfamiliar rats (matched body weights within 15 g) receiving the same pharmacological treatment, were placed in an unfamiliar open field arena and their behavior was recorded for 10 min in 1-min sections (Media Recorder, Noldus Information Technology, Wageningen, the Netherlands). All treatments and their corresponding vehicles were given 45 min prior to testing except EVP-6124 and risperidone, which were given 60 and 75 min prior to testing, respectively. The arenas were wiped with 20% ethanol between test sessions.

An experimenter blind to the treatments analyzed manually social interaction behavior for each pair of rats in the treatment groups using EthoVision XT v. 8.5 software (Noldus Information Technology). Sniffing the conspecific’s snout or parts of the body (including the anogenital region), following, walking around partner, climbing over or under, and mutual grooming were considered as social interaction whereas passive social contact or aggressive behavior were not. The locomotor activity of individual animals was analyzed automatically with EthoVision XT v. 8.5 software.

### Data analysis and statistics

A 7-min period between 3 and 10 min of each trial was analyzed (see the “Results” section). No animals were excluded from the data analysis. All values are presented as mean ± standard error of mean (SEM). Statistical analyses were performed using IBM SPSS Statistics software v. 21 (IBM Finland, Helsinki, Finland). The statistical significance of the differences between the treatments in social interaction and locomotor activity was assessed using one-way analysis of variance (ANOVA) followed by Tukey post-hoc test for group comparisons. The differences were considered to be statistically significant at the *p* < 0.05 level.

## Results

### Optimization of acute PCP dose inducing social interaction deficits

**Fig. 1:**
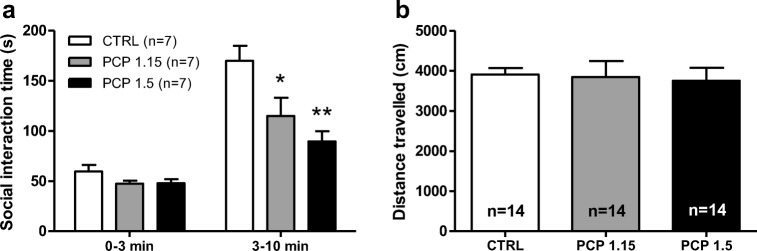
The effect of PCP (1.15 and 1.5 mg/kg s.c.) on the social interaction behavior between 0–3 min and 3–10 min (**a**) and locomotor activity between 3 and 10 min (**b**) in Wistar rats. Data are expressed as mean ± SEM. **p < 0.01, *p < 0.05 vs. controls (Tukey post hoc). CTRL control, PCP phencyclidine

**Fig. 2:**
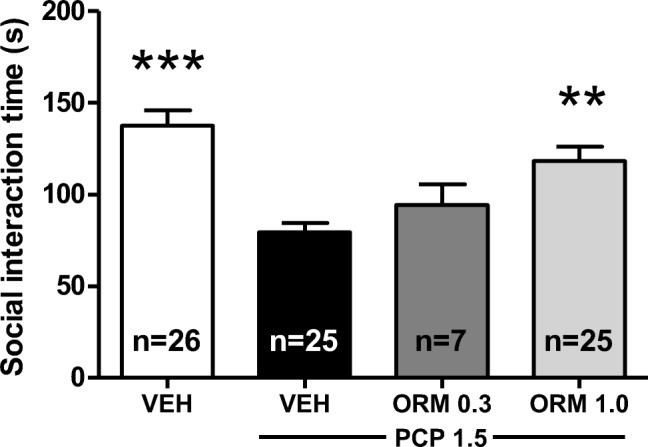
The effect of the adrenergic α_2C_ receptor antagonist ORM-13070 (0.3 and 1.0 mg/kg s.c.) on PCP-induced (1.5 mg/kg s.c.) social interaction deficits in Wistar rats. Data are expressed as mean ± SEM. ***p < 0.001, **p < 0.01 vs. PCP-treated group (Tukey post hoc). ORM ORM-13070, PCP phencyclidine, VEH vehicle

**Fig. 3:**
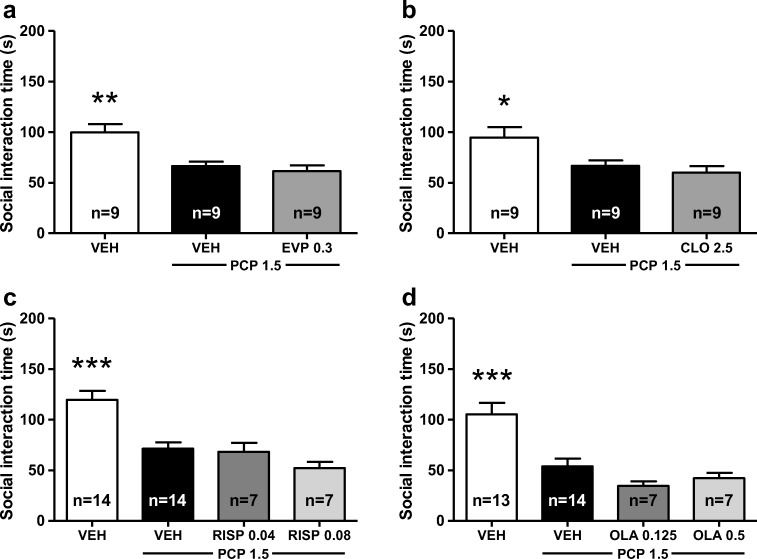
The effect of the α_7_ nicotinic acetylcholine receptor partial agonist EVP-6124 (0.3 mg/kg s.c.) (**a**), clozapine (2.5 mg/kg i.p.) (**b**), risperidone (0.04 and 0.08 mg/kg s.c.) (**c**), and olanzapine (0.125 and 0.5 mg/kg s.c.) (**d**) on PCP-induced (1.5 mg/kg s.c.) social interaction deficits in Wistar rats. Data are expressed as mean ± SEM. ***p < 0.001, **p < 0.01, *p < 0.05 vs. PCP-treated group (Tukey post hoc). CLO clozapine, EVP EVP-6124, OLA olanzapine, PCP phencyclidine, RISP risperidone, VEH vehicle

First, we optimized the acute PCP dose to induce social interaction deficits without affecting the locomotor activity of the rats. Interestingly, the initial minute-to-minute analysis of the behavioral data showed that there were no group differences in the social interaction times between PCP-treated (1.15 or 1.5 mg/kg) and control rats in the first 3 min of the 10-min test session (F_2,18_ = 2.02, *p* > 0.1) (Fig. 1a). Thus, to reduce the effects of nonspecific disturbing factors and to improve the sensitivity of the model, we examined only the time period between 3 and 10 min in all analyses. During this time period, acute PCP (1.15 and 1.5 mg/kg) significantly affected social interaction behavior (F_2,18_ = 7.89, *p* < 0.01) (Fig. 1a). The post-hoc analysis revealed that already the lower PCP dose 1.15 mg/kg significantly reduced the time spent in social interaction by 32% (*p* < 0.05); at the dose of 1.5 mg/kg, the effect was even more pronounced, i.e., a 47% reduction (*p* < 0.01) was observed compared to the control group. PCP administration had no effects on locomotor activity as indicated by the distance traveled during the social interaction task (F_2,39_ = 0.06, *p* > 0.9) (Fig. 1b). Since the PCP dose 1.5 mg/kg was found to induce a more marked defect on social interaction behavior without significantly affecting locomotor activity, we selected this dose for further testing with the experimental drugs and the antipsychotics. These subsequent experiments also confirmed that the PCP-induced deficits at the dose level of 1.5 mg/kg were robust and repeatable throughout the experiments (reduction of social interaction time by 29–49% compared to controls) (Figs. 2 and 3).

### ORM-13070 ameliorates acute PCP-induced deficits in social interaction behavior

After optimizing the PCP dose and modifying the social interaction study protocol, we examined the effects of an α_2C_ AR antagonist ORM-13070 on PCP-induced social interaction deficits. A significant overall difference was observed in the social interaction time after the treatments (F_3,79_ = 11.96, *p* < 0.001) (Fig. 2). The post-hoc analysis revealed that ORM-13070 1.0 mg/kg significantly ameliorated the PCP-induced deficits by increasing the social interaction time by 49% (*p* < 0.01). ORM-13070 0.3 mg/kg had no significant effect on PCP-induced social interaction deficits (*p* > 0.7) and no further studies were conducted with this dose. ORM-13070 had no effect on locomotor activity (Table 1).

**Table 1:** Locomotor activity (distance traveled in cm) in different treatment groups (data collected between 3 and 10 min in each 10-min trial). Doses are expressed as mg/kg. Data are expressed as mean ± SEM (number of animals per treatment group in parentheses).

	ORM-13070	EVP-6124	Clozapine	Risperidone	Olanzapine
Dose 1	0.3	0.3	2.5	0.04	0.125
Dose 2	1.0	n/a	n/a	0.08	0.5
SAL + Vehicle	4318 ± 162 (52)	4254 ± 141 (18)	4192 ± 180 (18)	3973 ± 107^*^ (28)	4517 ± 211^*^ (26)
PCP 1.5 + Vehicle	3777 ± 187 (50)	3617 ± 118 (18)	3871 ± 340 (18)	3161 ± 200 (28)	3379 ± 301 (28)
PCP 1.5 + Dose 1	3679 ± 516 (14)	4290 ± 296 (18)	1434 ± 199^***^ (18)	2612 ± 466 (14)	2997 ± 440 (14)
PCP 1.5 + Dose 2	3566 ± 171 (50)	n/a	n/a	1686 ± 221^***^ (14)	1756 ± 173^**^ (14)

^***^p < 0.001, ^**^p < 0.01, ^*^p < 0.05 vs. PCP 1.5-treated group (Tukey post hoc). *n/a* not available, *PCP* phencyclidine, *SAL* saline

The effect of ORM-13070 1.0 mg/kg on PCP-induced social interaction was assessed in three independent experiments to confirm the repeatability of the initial finding. These replicates confirmed that the effect of ORM-13070 was highly robust and repeatable, since a significant overall difference was observed in social interaction time after the treatments in all independent experiments (F_2,18_ = 13.75, *p* < 0.001; F_2,25_ = 8.89, *p* < 0.01; and F_2,24_ = 6.06, *p* < 0.01, respectively), and the post-hoc analysis revealed that ORM-13070 at 1.0 mg/kg dose significantly ameliorated the PCP-induced deficits by increasing the social interaction time by 53% (*p* < 0.05), 53% (*p* < 0.05), and 40% (*p* < 0.05), respectively, compared to the corresponding PCP groups.

### EVP-6124 and atypical antipsychotics have no effect on the acute PCP-induced deficits in social interaction behavior

Next, we compared the effects of the α_2C_ AR antagonist ORM-13070 to those of an α_7_ nAChR partial agonist EVP-6124, and to three atypical antipsychotics clozapine, risperidone, and olanzapine. Neither EVP-6124 nor any of the three tested antipsychotics were able to reverse the PCP-induced deficits in the social interaction behavior (Fig. 3a–d). In confirmation of the above findings, the main effect of the treatment was observed, as PCP administration significantly reduced the social interaction time in all four experiments (EVP-6124: F_2,24_ = 11.16, *p* < 0.001, Fig. 3a; clozapine: F_2,24_ = 5.67, *p* < 0.01, Fig. 3b; risperidone: F_3,37_ = 13.19, *p* < 0.001, Fig. 3c; olanzapine: F_3,37_ = 12.92, *p* < 0.001, Fig. 3d). However, the post-hoc analysis revealed that none of the compounds affected the PCP-induced reduction in the social interaction time (EVP-6124 0.3 mg/kg: *p* > 0.8; clozapine 2.5 mg/kg: *p* > 0.8; risperidone 0.04 mg/kg: *p* > 0.9; risperidone 0.08 mg/kg: *p* > 0.4; olanzapine 0.125 mg/kg: *p* > 0.4; olanzapine 0.5 mg/kg: *p* > 0.8). Clozapine 2.5 mg/kg and the higher doses of risperidone (0.08 mg/kg) and olanzapine (0.5 mg/kg) significantly decreased locomotor activity compared to the corresponding PCP groups by 63% (clozapine: F_2,51_ = 36.38, *p* < 0.001), 47% (risperidone: F_3,80_ = 16.12, *p* < 0.001), and 48% (olanzapine: F_3,78_ = 13.62, *p* < 0.01), respectively (Table 1).

## Discussion

PCP is commonly used to induce schizophrenia-like social interaction deficits, but no validated acute low dose PCP administration protocols that could be used for drug screening purposes in early drug development have been published. Therefore, we first modified a feasible social interaction rat model based on acute PCP administration for the assessment of the negative schizophrenia-like symptoms (Sallinen et al. 2013a). Single PCP doses between 1.0–2.0 mg/kg have been used to induce social interaction deficits in rats (Corbett et al. 1995; Boulay et al. 2004; Savage et al. 2011; Sallinen et al. 2013a). However, PCP already at a dose of 2.0 mg/kg has been shown to induce nonspecific effects, such as stereotypy, ataxia and hyperactivity, mimicking some of the positive symptoms of schizophrenia and also affecting the social interaction of the animals (Castellani and Adams 1981; Sams-Dodd 1996; Boulay et al. 2004). These nonspecific effects were recently verified also by our functional MRI study which revealed the diffuse disrupting effects of PCP on rat brain connectivity at doses ≥ 2.0 mg/kg (Paasonen et al. 2017). In the present study, both selected PCP doses (1.15 and 1.5 mg/kg) significantly reduced the time that the rats spent in social interaction but had no effect on locomotor activity, suggesting that the selected doses of PCP were able to mimic the social withdrawal characteristic of schizophrenia. Thus far, acute single PCP doses lower than 1.5 mg/kg have been successfully used only in experimental set-ups with a reversed light-dark cycle (Sams-Dodd 1996; Boulay et al. 2004; Sallinen et al. 2013a). In our study, a normal light-dark cycle and no preceding habituation to the test environment were used in order to increase the throughput of the test. The improved sensitivity of the present study protocol in detecting social withdrawal under a normal light-dark cycle may be explained by the fact that only the time period between 3 and 10 min was used in the data analysis. Our minute-to-minute analysis revealed that the initial habituation to the new test environment disturbed the social interaction behavior during the first minutes of the experiments. By excluding the first 3 min from each trial, the test measured more specifically the social interaction behavior, thus increasing the test’s sensitivity.

Next, we compared the effects of a selective α_2C_ AR antagonist and various pharmacotherapies on the social interaction behavior. The α_2C_ AR antagonist ORM-13070 significantly ameliorated acute PCP-induced social interaction deficits, whereas, the α_7_ nAChR partial agonist and three atypical antipsychotics were ineffective. The favorable effect of the α_2C_ AR antagonist, ORM-13070, is in accordance with earlier studies conducted with another α_2C_ AR antagonist, ORM-10921, which was able to totally reverse the acute PCP-induced social interaction deficits in rats (Sallinen et al. 2013a). Furthermore, it has been shown that α_2C_ AR antagonists ameliorate also positive and cognitive deficits associated with schizophrenia in PCP-induced and neurodevelopmental rat models (Sallinen et al. 2007, 2013a; Uys et al. 2016). ORM-12741, another highly selective α_2C_ AR antagonist (α_2C_/α_2A_ ratio 100) pharmacologically closely resembling ORM-13070, has entered clinical trials assessing its effects on cognitive functions (Sallinen et al. 2013b; Rinne et al. 2017). As far as we are aware, however, the efficacy of this compound on social interaction deficits has not been evaluated. Nevertheless, such studies would be highly interesting since the clinical trials have shown that ORM-12741 can be safely administered to humans. One possible mechanism to account for the ability of α_2C_ AR antagonists to exert effects on negative symptoms might be the modulation of the firing activity of the prefrontal dopaminergic neurons originating from the ventral tegmental area (Sallinen et al. 2013a). The hypofunction in these neurons has been closely associated with pronounced negative symptoms in schizophrenia patients (Winograd-Gurvich et al. 2006; Schwartz et al. 2012), and the functional connectivity of the prefrontal cortical area was significantly changed by acute low PCP dose (2.0 mg/kg s.c.) (Paasonen et al. 2017). The α_2C_ antagonist ORM-10921 increased dopamine release in the medial prefrontal cortex of rats (Sallinen et al. 2013a), which could partly explain the beneficial effects of α_2C_ AR antagonists on PCP-induced cortical dysfunction. On the other hand, α_2C_ ARs might directly contribute to the regulation of DA release at the terminal level in the medial prefrontal cortex (Ihalainen and Tanila 2002). Furthermore, α_2C_ AR antagonists may modify striatal GABAergic output (Holmberg et al. 1999; Zhang and Ordway 2003), thus affecting multiple transmitter systems contributing to dopamine turnover in prefrontal cortical regions. However, the precise mechanism by which α_2C_ AR antagonists exert their effects remains to be resolved.

This is the first study to assess the effects of the α_7_ nAChR partial agonist, EVP-6124, in acute PCP-induced social interaction deficits in rats. We detected no effect at selected dose, although EVP-6124 and a full α_7_ nAChR agonist, TC-5619, have been reported to be able to reverse subchronic PCP-induced deficits in social interaction behaviors in mice (Pedersen et al. 2014). Furthermore, type I and type II α_7_ nAChR PAMs together with an orthosteric agonist and partial agonist have all alleviated acute ketamine-induced social interaction deficits in rats (Nikiforuk et al. 2016; Potasiewicz et al. 2017). This discrepancy can be explained by the differences in experimental models and the use of various NMDA antagonists. In addition, a rat in vivo microdialysis study investigating the effects of EVP-6124 on various neurotransmitter levels found evidence for an inverted U-shaped dose-response profile for EVP-6124 (Huang et al. 2014), suggesting that the dose-responsiveness of EVP-6124 may vary between models. Therefore, more in-depth studies should be undertaken to investigate the dose-responsiveness of EVP-6124 in NMDA antagonist-induced social interaction model.

Atypical antipsychotics, such as clozapine, risperidone and olanzapine, have shown some efficacy in reversing social interaction deficits in rat models (e.g., Corbett et al. 1995; Sams-Dodd 1996, 1997) although also opposite results have been reported (e.g., Corbett et al. 1995; Sams-Dodd 1997; Boulay et al. 2004; Sallinen et al. 2013a). Classical antipsychotics, which act mainly as dopamine D_2_ receptor antagonists, have no effect on PCP-induced social interaction deficits (e.g., Corbett et al. 1995; Sams-Dodd 1996; Boulay et al. 2004; Bruins Slot et al. 2005). These contradictory results agree with clinical studies indicating that atypical antipsychotics, acting through multiple receptor systems in addition to dopaminergic D_2_ receptors (e.g., serotonergic, adrenergic, and cholinergic), ameliorate the negative symptoms of schizophrenia, although their efficacy is far from satisfactory (Kirkpatrick et al. 2006; Hanson et al. 2010; Sarkar et al. 2015). As there is no drug of choice for the treatment of negative symptoms in patients, no true positive control compound exists. Thus far, it is not known which mechanism of the multimodal effects of atypical antipsychotics accounts for their efficacy against the negative symptoms of schizophrenia. Notably, α_2C_ AR antagonism is a common characteristic of certain atypical antipsychotics postulated to have beneficial effects on the negative symptoms, and especially, a high antagonistic α_2C_/D_2_ ratio, seems to be favorable (Kalkman and Loetscher 2003; Brosda et al. 2014). The three antipsychotics tested in the present study all have higher affinity for the α_2C_ ARs than for the α_2A_ ARs (α_2C_/α_2A_ ratios: clozapine 4.2; risperidone 116; olanzapine 16.2) (Brosda et al. 2014). However, only clozapine has a α_2C_/D_2_ ratio ≥ 1, which is considered necessary if the α_2C_ AR antagonism is to possess any clinical relevance (Kalkman and Loetscher 2003).

The effects of atypical antipsychotics on NMDA antagonist-induced deficits in social interaction behavior in preclinical schizophrenia models are variable and seem to be dependent on several factors, e.g., on the dosing regimen of the drugs and the applied NMDA antagonist. For example, acute clozapine appears to be relatively ineffective in PCP models (Sams-Dodd 1998; Boulay et al. 2004; Bruins Slot et al. 2005; Sallinen et al. 2013a, however, see Corbett et al. 1995), but subchronic administration may exert some positive effects (Sams-Dodd 1996, 1998). Similarly, risperidone and olanzapine lack efficacy against PCP-induced social interaction deficits (Corbett et al. 1995; Sams-Dodd 1997; Boulay et al. 2004; Pedersen et al. 2014) although some positive results, especially with higher or repeated doses of risperidone, have also been reported (Corbett et al. 1995; Sams-Dodd 1997; Pouzet et al. 2002). We also tested higher doses of risperidone and olanzapine (0.08 mg/kg s.c. and 0.5 mg/kg s.c., respectively), but they both tended to further reduce the social interaction time and substantially decreased the locomotor activity of the PCP-treated rats (Table 1). As clozapine significantly decreased the locomotor activity already at the tested dose without affecting social interaction behavior, higher doses were not tested. One can hypothesize that significant α_2_ antagonism and reversal of PCP-induced social interaction deficit without undesired motor side effects is difficult to achieve by acute clozapine. In fact, pharmacokinetic modeling data by Li et al. (2014) indicates that predicted α_2_ occupancy is only approximately 30% at plasma clozapine levels bringing > 90% muscarinic M_1_ and histamine H_1_ occupancy and approximately 50% D_2_ occupancy. Interestingly, an antipsychotic drug, aripiprazole, has shown some efficacy in preclinical studies against PCP-induced social interaction deficits (Bruins Slot et al. 2005; Snigdha and Neill 2008). However, in the study of Tarland et al. (2018), aripiprazole was unable to reverse the social interaction deficits. Aripiprazole has strikingly different receptor binding profile compared to atypical antipsychotics used in this study, including agonistic effect on D_2_ receptors and relatively high affinity for serotonergic 5-HT_1A_ receptors, which may explain its positive effects on social interaction behavior (Bruins Slot et al. 2005; Snigdha and Neill 2008). Altogether, our data agree with clinical findings where atypical antipsychotics have shown conflicting effects on the negative symptoms of schizophrenia, and furthermore, subchronic treatment is required in order to reveal their antipsychotic properties (Kirkpatrick et al. 2006; Sarkar et al. 2015). Thus, more work will be required to establish the effects of subchronically administered antipsychotics in the present social interaction test protocol.

In conclusion, an acute low dose PCP induces highly robust and repeatable schizophrenia-like social interaction deficits in Wistar rats. Most importantly, we confirmed that a selective α_2C_ AR antagonist, ORM-13070, could ameliorate the PCP-induced social interaction deficits in rats, supporting the hypothesis that α_2C_ AR antagonism is a potential mechanism for the treatment of the negative symptoms of schizophrenia.
